# Application of inverse gas chromatography in the surface characterization of diethanol amine modified polystyrene based polymer

**DOI:** 10.3906/kim-2105-45

**Published:** 2021-10-19

**Authors:** Ayşegül Çiğdem ADIGÜZEL, Burak KORKMAZ, Fatih ÇAKAR, Bahire Filiz ŞENKAL, Özlem CANKURTARAN

**Affiliations:** 1 Department of Chemistry, Faculty of Engineering and Natural Sciences, Bursa Technical University, Bursa Turkey; 2 Department of Chemistry, Faculty of Science and Letters, İstanbul Technical University, İstanbul Turkey; 3 Department of Chemistry, Faculty of Arts and Sciences, Yıldız Technical University, İstanbul Turkey

**Keywords:** Inverse gas chromatography, surface properties, polystyrene based polymer

## Abstract

In the present work, diethanol amine modified polystyrene based polymer (PVBC-Diethanol amine) was synthesized and characterized, then surface properties of the polymer were examined by inverse gas chromatography method at infinite dilution. The retention diagrams obtained based on the interaction of polar and nonpolar probes with the polymer were drawn over a temperature range from 30 to 55 °C. Through the diagrams, the dispersive component of the surface free energy, g*_S_* ^D^ of the polymer surface, and the specific enthalpy of adsorption, D*H* *_A_* *^S^* , of probes on the polymer were also calculated. Lewis acid, *K* *_A_*, and Lewis base, *K* *_D_* , parameters of PVBC-Diethanol amine surface were determined. The values of *K* *_A_* and *K* *_D_* indicated that PVBC-Diethanol amine surface exhibited a basic behavior.

## 1. Introduction

The inter- and intra- molecular interactions of polymers determine their physicochemical behavior and play a key role in the design of new materials and processes. However, the long-chain character of polymers, in comparison to low molecular weight compounds, poses a few fundamental problems when trying to describe their intermolecular interaction, for example, in interface environments [1]. 

Inverse gas chromatography (IGC) [2–3] is a precise and reliable method that has been used for over 40 years to determine polymer properties by investigating polymer interactions with low molecular weight compounds [4]. The method is also economical as it requires a limited amount of probes and polymer samples.

In the IGC method, the material such as polymer and polymer mixtures [5] to be examined is placed on a column as a stationary phase, which is different from the common gas chromatography (GC) of the IGC method. Probes (solvents) of known properties are then injected into the chromatographic column containing the polymer. Retention times for probes passing in the column are used to describe their interactions with the investigated material.

The activity of a material’s surface depends on the nature of the surface, such as surface free energy and acidity-basicity [6–7]. It is possible to obtain information about many important surface properties of polymers such as the dispersive component of surface free energy [7–9], specific (acid-base) interactions [10–12], thermodynamic parameters of adsorption/sorption [13–14] for polymeric systems over a wide temperature range by the IGC. 

Since its discovery, polystyrene has brought its recognition and usefulness to a whole new level. In addition to being widely known, it has been frequently demonstrated that polystyrene and its derivatives are reliable and always open to innovation, with their application areas, wide areas of use, and postsynthesis modification [15]. Vinyl benzyl chloride, a known member of the styrene family, is at this point open to postpolymerization modifications because of the convenience provided by the activity of the methyl chloride group [16]. The polymer chain to be synthesized from this monomer forms a suitable backbone for the development of many applicable materials, from adsorption to the production of materials to be used in display technologies [17], selectivity for certain ions [18] or development of ion exchange membranes [19], through modifications to be made with the required functional groups. In addition to these areas of use, these properties have led to the emergence of different application areas with new developments that enable the development of different surface area properties [20–22]. Not only the homopolymers but also the copolymers to be produced contribute to the diversity in these application areas [18–20]. At this point, in this study, it has been shown again that it is an especially useful material for postpolymerization modification. 

Synthesis of the diethanol amine modified polystyrene based monomer, and its polymer were described in the literature [23]. In this study, poly (vinyl benzyl chloride) (PVBC) was synthesized by free radical polymerization method. The polymer was reacted with diethanol amine to obtain PVBC-Diethanol amine (PVBC-DEA) and characterization of PVBC-DEA was carried out. The surface properties of PVBC-DEA were investigated using the IGC method at infinitely dilute conditions. 

## 2. Inverse gas chromatography theory for surface characterization

While examining the properties of polymers with IGC experiments, the probe-probe interactions are neglected by working under infinitely dilute conditions and only polymer-probe interactions are considered. In the method, the polymer sample examined as a stationary phase is placed in the chromatographic column, selected probes are injected into the column, and the retention times of the probes in the column are recorded. There is a relationship between polymer-probe interactions and a net retention volume, *V* *_N_*, given by the following equation:

(1)VN=Q.J.(tR-tA).(T/Tr)

Where *Q* is volumetric flow rate measured at room temperature; *J* is James-Martin gas compressibility correction factor term; *t* *_R_* is retention time of the probe and *t* *_A_* is retention time of air; *T* and *T* *_r_* are temperature of the studied column and room temperature, respectively [24].

Surface free energy of the polymer: The surface of solids can be defined by the dispersive or specific component of their surface free energy. These components allow describing the surface as electron acceptor or an electron donor to express the surface properties numerically. The surface free energy is given by equation (2) [25]; 

(2)γs=γsD+γss

The dispersive component, g*_S_* ^D^, defines van der Waals dispersion forces of the solid, while the specific component, g*_S_* ^D^ , includes all polar forces such as acid-base, H-bond, and dipole forces. Dorris–Gray and Schultz methods can be used to obtain the g*_S_* ^D^ values.

According to the method proposed by Dorris and Gray [26] to determine g*_S_* ^D^ value is given by equation (3).

(3)ΔGA[CH2]=2NAa[CH2]γsDγL[CH2]

where, D*G* *_A[CH2]_* is the adsorption free energy for a methylene group; *N* *_A_* is Avogadro constant; *a* *_[CH2]_* is the surface area of an adsorbed methylene group and g*_L[CH2] _* is the surface free energy of a solid constituted solely by methylene groups, such as polyethylene g*_L[CH2]=_* 35.6 - 0.058(t), is the operating temperature. D*G* *_A[CH2]_* is obtained experimentally according to equation (4). 

(4)Δ GA[CH2]=-RT ln(VN,nVN,n+1

Where *V* *_N,n_* is the retention volume of n-alkanes containing n carbon atoms while *V* *_N,n+1_* is the retention volume of n-alkanes containing n+1 carbon atoms. 

For an n-alkane probe series, the plot of the carbon numbers of n-alkanes versus *RTlnV* *_N_* values is linear. D*G* *_A[CH2]_* value is found from the slope of the linear line. And the values of g*_S_* ^D^ can be determined by equations (3) and (4).

The free energy of adsorption, D*G* *_A_*, can be obtained by the following equation using net retention volume data [27]:

(5)ΔGA=-RT lnVN+K

Where *K* is a constant. 

Schultz realized that g*_S_* ^D^ values obtained by the method he developed were compatible with the Dorris-Gray method. According to this method, g^D^ is given by the following equation [27]

(6)-ΔGA=RT lnVN=2NAaγSDγLD+K''

Where g*_L_* ^D^ is the dispersive component of the surface free energy of the adsorbent and *K* *^”^* is a constant. It is possible to calculate the g*_S_* ^D^ value of the polymer at the experiment temperature from the slope of the *RTlnV* *_N_* *– a(*g*_L_* ^D^ *)* *^0.5 ^* graph, using n-alkane probe series. The values of *a(*g*_L_* ^D^ *)* *^0.5^* are obtained from the literature [27].

Acid-base properties of the polymer: A polar probe in the IGC column interacts both specifically and dispersively with the stationary phase. Specific interactions refer to all other interactions such as polar, H-bond, metallic or magnetic, which are interactions other than dispersive interactions [28]. Hence, a specific component of the adsorption free energy, D*G* *_A_* *^S^* , can be defined by equation (7).

(7)ΔGA=ΔGAS+ΔGAD

Where D*G* *_A_* *^D^* is the dispersive component of the adsorption free energy. D*G* *_A_* *^S^*, is defined by the following equation.

(8)-ΔGAS=RT ln(VN,nVN,ref)

Where *V* *_N_* is the retention volume for the polar probe and *V* *_N,ref_* is the retention volume for n-alkanes reference line. *-*D*G* *_A_* *^S ^* , is determined by the distance between the ordinate values of the polar probe data point and the n-alkane reference line on the *RTlnV* *_N_* *– a(*g*_L_* ^D^ *)* *^0.5^* graph [29]. 

Adsorption of a polar probe to an adsorbent surface changes both the enthalpy and entropy of the system. Equation (9) defines the relationship between enthalpy, entropy values and D*G* *_A_* *^S^* values.

(9)δGAS=δAS-T.ΔSAS

where, D*H* *_A_* *^S^* is the adsorption enthalpy, D*S* *_A_* *^S^* is the adsorption entropy; *T* is the studied column temperature. D*H* *_A_* *^S^* and D*S* *_A_* *^S^* values are found by plotting -D*G* *_A_* *^S^* */ T* versus *1/T* values using equation (10), for polar probes.

(10)ΔGAS/T=ΔHAS/T-ΔSAS

D*H* *_A_* *^S^* and D*S* *_A_* *^S^* are related to Lewis acid-base properties [29]. The D*H* *_A_* *^S^* value is due to specific interactions between the adsorbent surface and the polar probe, and is associated with the acidic-basic character as seen in equation (11).

(11)-ΔHAS/AN*=KA.DN/AN*+KD

Where *DN* is Gutmann’s donor; AN* is Gutmann’s modified acceptor numbers [29]. *K* *_A_* represent Lewis acidity of the adsorbent surface while *K* *_D_* represent basicity of the adsorbent surface. According to equation (19), when *-*D*H* *_A_* *^S^* / AN*  values are plotted against DN / AN* the slope and intercept of the line are equal to *K* *_A_* and *K* *_D _*, respectively. The ratio of *K* *_D_* and *K* *_A_* values to each other gives information about the acidic-basic character of the surface. Accordingly, the condition for the surface to be considered acidic is *K* *_D_* */ K* *_A_* *< 1* and the condition to be considered basic is *K* *_D_* */ K* *_A_* *> 1*.

## 3. Materials and instrumentation

Chromosorb-W (AW-DMCS-treated, 80/100 mesh) used as support material for the chromatographic column were supplied by Merck AG Inc., Germany. Silane-treated glass wool which was used to plug the ends of the chromatographic column was provided by Alltech Associates Inc., USA. Abbreviation, source, mass fraction purity, and CAS registry number of all probes in used the IGC experiments and other chemicals used in the synthesis are given in Table 1.

**Table 1:** Abbreviation, source, mass fraction purity, and CAS registry number of the chemicals.

Chemical name	Abbreviation	Source	Purity	CAS number
n-hexane	Hx	Supelco	≥ 0.990	110-54-3
n-heptane	Hp	Merck	≥ 0.990	142-82-5
n-octane	O	Merck	≥ 0.990	111-65-9
n-nonane	N	Merck	≥ 0.990	111-84-2
n-decane	D	Merck	≥ 0.995	124-18-5
Ethyl acetate	EA	Merck	≥ 0.995	141-78-6
Acetone	Ace	Supelco	≥0.998	67-64-1
Dichlorometane	DCM	Supelco	≥0.995	75-09-2
Trichlorometane	TCM	Supelco	≥0.990	67-66-3
Tetrahydrofurane	THF	Supelco	≥0.998	109-99-9
Diethanolamine	-	Sigma-Aldrich	≥0.980	111-42-2
Vinylbenzyl chloride	VB	Sigma-Aldrich	≥0.970	30030-25-2

IGC experiments were performed on Agilent Technologies 6890N model gas chromatography device equipped with a thermal conductivity detector,* USA.* The IGC column used was purchased from Alltech Associates, Inc., *USA*, was made from a stainless-steel tube (3.2 mm o.d. and 1 m length).

To prepare the PVBC-DEA coated support material, firstly the polymer sample was dissolved in DMSO solvent, and then Chromosorb W solid was added to the obtained polymer solution. The slow evaporation method was used to remove the DMSO solvent from the solution.

The prepared material (1.326* g*) was filled into the chromatographic column and conditioned under helium (He) atmosphere for 24 *h* at 373.2 *K*. Probes used were injected into the column using a 1 *μL* Hamilton syringe, Romania. During each injection procedure, 1 *μL* of probe was poured into the syringe, and then the remaining portion of the syringe was filled with air. The average values of retention times were determined by taking at least consecutive probe injections at each column temperature studied. These values were used for reported values of retention volumes, *V* *_N_* The percent error in *V* *_N_* was calculated as less than ± 0.5. Experimentally, the flow rate for the carrier gas (He) was decided as 3-4 *cm* *^3^* *. min* *^-1^*. And was kept constant throughout the IGC measurements. 

A TA/Discovery DSC251 model DSC device and a Thermo Scientific Nicolet 380 Spectrometer device were used for the analysis of the polymers obtained.

## 4. Result and discussion

### 4.1. Synthesis and modification with diethanol amine of poly (vinyl benzyl chloride) (PVBC)

**Figure 1:**
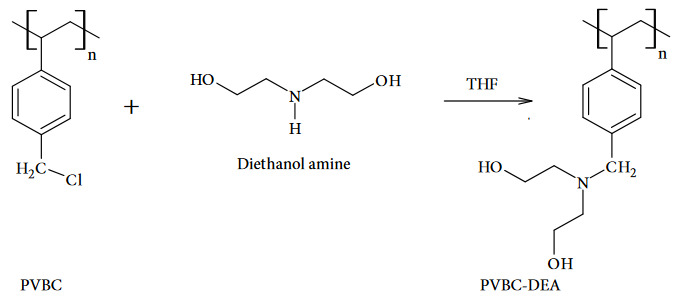


For the synthesis of PVBC, free radical polymerization method was used as it is described in literature [30]. 10 mL (0.071 mol) of Vinyl benzyl chloride and 0.155 g (0.95 mmol) of AIBN were dissolved in 40 mL of pre-dried THF in a two-necked round-bottom flask. The polymerization was performed under a nitrogen atmosphere at 65 ^o^C for eight hours. After the reaction was terminated, the resulting viscous mixture was allowed to cool to room temperature. After that, the polymer solution was slowly precipitated in hexane dropwise and the precipitated polymer was purified by filtration. The resulting polymer was washed with excess Hx and dried overnight under a vacuum. 9.5 g of PVBC (Figure 1) were obtained.

For the postpolymerization modification reaction, 1 g of synthesized PVBC and 1.1 g (7.8 mmol) of potassium carbonate as an acid scavenger were dissolved in predried 15 mL of THF in a reaction flask and stirred in this manner for twenty minutes. The reaction flask, which continued to stir, was placed in an ice bath at 0 ^o^C . Solution of 0.8 mL (7.8 mmol) of diethanol amine dissolved in 5 mL of dried THF, was added dropwise to the reaction vessel. After the completion of the addition, reaction was stirred for 18 hours at room temperature. Subsequently, the reaction was stirred for 3 hours more but at 60 ^o^C . Lastly, the reaction was terminated and the mixture, which was let to cool to room temperature, was precipitated dropwise in hexane and purified, and then filtered. The resulting polymer was dried under a vacuum overnight. Obtained diethanol amine modified PVBC (Figure 1) was weighed as 0.90 *g*.

0.10 *g* of diethanol amine modified PVBC was added to 10 mL of 0.10 M HCl solution. This mixture was stirred and dissolved at room temperature. Then, 3 mL of filtrate was titrated with 0.10 M NaOH solution using phenolphthalein as a colour indicator. At the end of titration result, total amine content of the polymer was calculated as about 4.50 mmol/g polymer.

**Figure 2:**
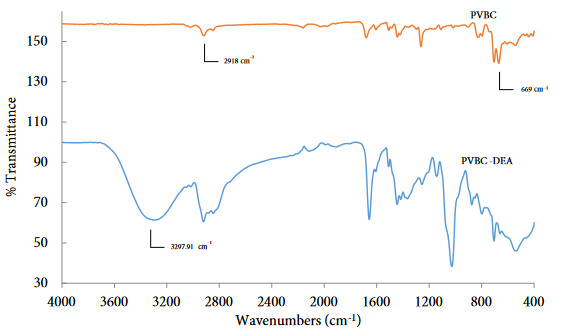


FT-IR spectroscopy was used for characterizations of synthesized and modified polymers. As it is seen in Figure 2, the difference between PVBC and modified-PVBC spectrums can be detected around at 1365.43 cm^–1^ and 1254.95 cm^–1^, which are belongs to C-N stretching of newly bonded benzyl carbon and nitrogen of diethanol amine. In addition, the stretching peaks of the methylene and methyl groups of the polymer at 2918 cm^–1^ and the C-Cl stretching peak at 669 cm^–1^, which is observed to disappear in the spectrum of the modified polymer but is seen in the main structure, evidence confirms the accuracy of the structures.

### 4.2. Surface characterization of PVBC-DEA

**Figure 3:**
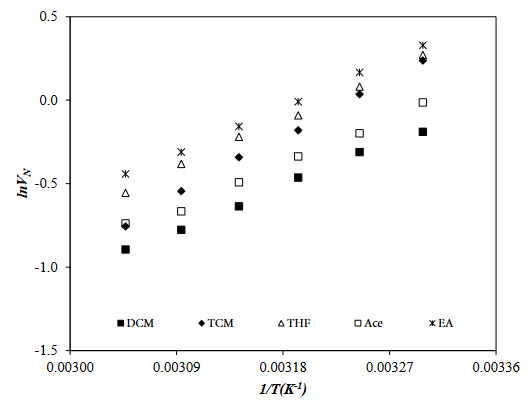


**Figure 4:**
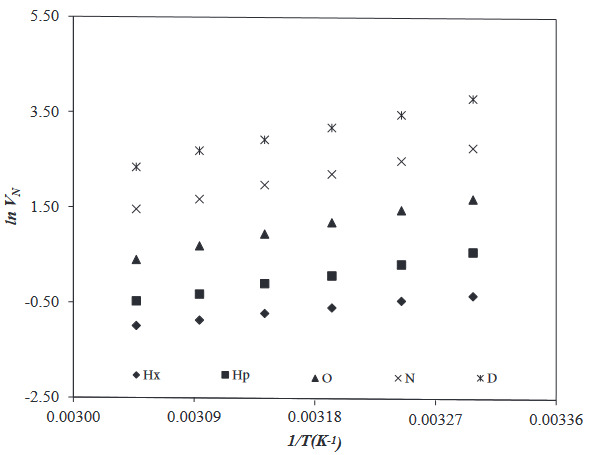


To investigate the adsorption, surface, and Lewis acid-base properties of the PVBC-DEA, EA, Ace, DCM, TCM, THF (polar probes), and Hx, Hp, O, N, and D (nonpolar probes) was passed through the IGC column in the temperature range 30 to 55 ^o^C. The net retention volume, *V* *_N _*, of the probes used on the polymer were calculated from equation (1) and the retention diagrams drawn are presented in Figures 3 and 4. The dispersive component of the surface free energy, g*_S_* ^D^, of PVBC-DEA was obtained from both Dorris–Gray method and Schultz method using equations (3) and (6), respectively.

**Figure 5:**
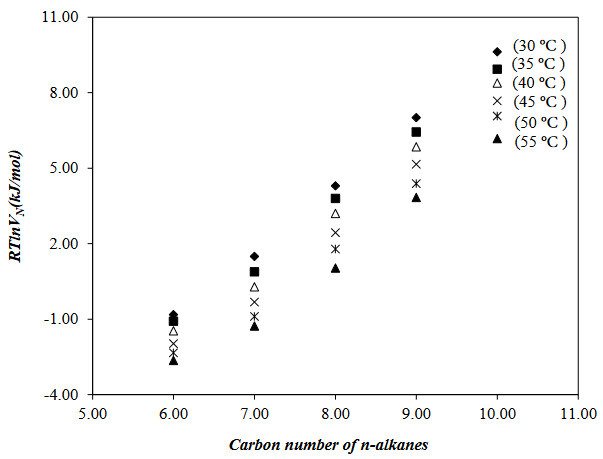


According to Dorris–Gray method, the adsorption free energy of one methylene group, D*G* *_A[CH2]_*, was determined from the slope of the lines plotted between the carbon number of alkanes and *RTlnV* *_N_* in the studied temperature range (Figure 5). g*_S_* ^D^ values were calculated by equation (3) and are presented in Table 2.

**Table 2:** The obtained values from Dorris–Gray method and Schultz method for PVBC-DEA at studied temperatures.

t (oC)	Dorris–Gray method	Schultz method
	gSD (mj/m2)	gSD (mj/m2)
30	39.43	37.94
35	37.37	35.65
40	36.72	34.72
45	35.61	33.40
50	33.99	31.58
55	30.55	28.16

**Figure 6:**
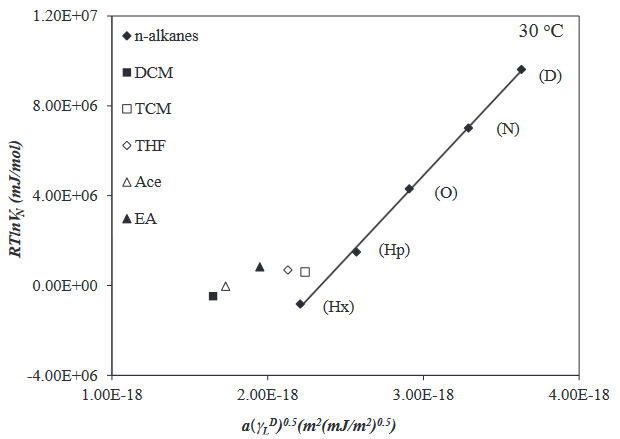


Using by Schultz method, the *a(*g*_L_* ^D^ *)* *^0.5^* *- RTlnV* *_N_* graph was plotted in the 30 to 55 ^o^C temperature range for n-alkanes and polar probes (for example the graph at 30 ^o^C is in Figure 6). The g*_S_* ^D^ values found from the slope of the reference line of alkanes in the graphs drawn according to the method are presented in Table 2. 

As it can be observed from Table 2, It has been determined that the g*_S_* ^D^ values found by both methods are remarkably close to each other and decrease with increasing temperature. An increase in temperature lowered the values of g*_S_* ^D^ significantly which may be caused by the expansion of the surface at higher temperatures [31]. Generally, there is a linear relationship between the variation of g*_S_* ^D^ and temperature. Therefore, its value at room temperature can be obtained using the extrapolation method [32]. In [32], by extrapolation, g*_S_* ^D^ values of polystyrene were found 23.41 mj/m^2^ for 25 ^o^C using Dorris-Gray method and 22.70 mj/m^2^ for 25 ^o^C using Schultz method. We report the g*_S_* ^D^ values for polystyrene based polymer PVBC-DEA as 41.18 mj/m^2^ for 25 ^o^C using Dorris-Gray method, and 39.82 mj/m^2^ for 25 ^o^C using Schultz method.

It is known that most polymeric materials have g*_S_* ^D^ values between 20 and 40 mj/m^2^ [33]. The g*_S_* ^D^ values of PVBC-DEA are consistent with this information. 

The values of D*H* *_A_* *^S^* for polar probes were determined from equations (8) and (10) and were presented in Table 3. According to Table 3, It has been observed that the value of D*H* *_A_* *^S^* increases in the following order: TCM>DCM>Ace>EA>THF. The THF as a probe molecule (*DN* = 84.4, *AN* = 2.1) exhibited the lowest -D*H* *_A_* *^S^* value, which is to be expected considering the basic properties of this molecule and the basic properties of PVBC-DEA surface given by the *K* *_D_* value. TCM is an acidic probe molecule (*DN* = 0.0, *AN* = 22.7), it may be expected to interact strongly with basic surfaces. 

**Table 3:** The values of -DHAS of adsorption on PVBC-DEA for the polar probes.

Polar probe	EA	Ace	DCM	TCM	THF
-ΔHAS (kJ/mol)	4.84	6.74	7.41	7.53	3.31

**Figure 7:**
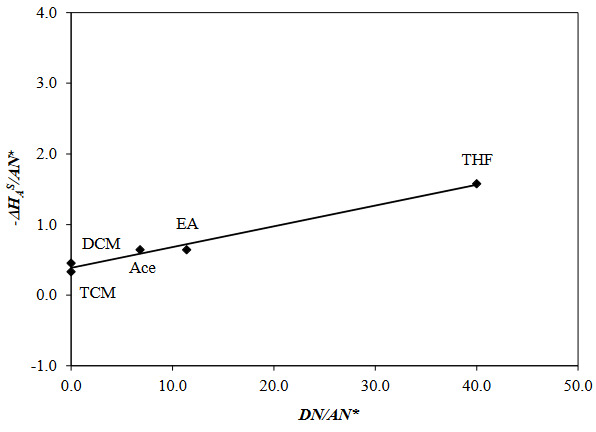


The values of *K* *_A_* and *K* *_D_* for the surface of PVBC-DEA were calculated by the graph (Figure 7), drawn using equation (11). In the literature [33], by IGC experiments, *K* *_D_* */K* *_A _* for polystyrene sample was determined as [(1.66)/(0.21)]>1, and the character of its surface is defined as basic. In this study, *K* *_D_* */K* *_A_* [(0.412)/(0.029)]>1, it is understood that the polymer surface exhibits a basic behavior. 

## 5. Conclusion

In this work, we first performed the synthesis and characterization of polystyrene-based polymer PVBC-DEA modified with dibutyl amine. Then, by IGC method, we investigated the interaction of various nonpolar and polar probes with the polymer surface. We determined the g*_S_* ^D^ values for PVBC-DEA as 30.55–39.43 mj/m^2^ using the Schultz method and 28.16–37.94 mj/m^2^ using the Dorris-Gray method. It is understood that an increase in temperature from 30 to 55 ^o^C causes a decrease in the g*_S_* ^D^ values found by both methods. We found the *K* *_A_* and *K* *_D_* values to be 0.029 and 0.412, respectively. Since the *K* *_D_* value is higher than the *K* *_A_*, the PVBC-DEA surface can be said to have a basic character. According to the experimental results obtained, it has been understood that IGC is a suitable method to examine the surface properties of the polymer.
